# New developments in the pathology of malignant lymphoma: a review of literature published from January 2015 to April 2015

**DOI:** 10.1007/s12308-015-0249-0

**Published:** 2015-06-17

**Authors:** J. Han van Krieken

**Affiliations:** Department of Pathology, Radboud University Medical Centre, P.O. Box 9101, 6500, HB Nijmegen, The Netherlands

## Introduction

The amount of data we need to digest becomes almost too large: data from large sequencing experiments and also data from the increasing number of scientist around the world. Looking at the series of articles, I had to go through for this review I sometimes feel overwhelmed. Nevertheless, I have tried to make my personal selection again.

## Biology of lymphoma

The possibilities of generating large amounts of data are enormous indeed. The challenge is to generate meaningful information from the results of large sequencing approaches. A nice example is the approach by Cimmino et al. [1]. They focus on ‘ten-eleven translocation 1’ (TET1) which is an important regulator of 5-hydroxymethylcytosine (5hmC) in embryonic stem cells, and the loss of 5hmC in many tumours suggests a critical role for the maintenance of this epigenetic modification. In mice, they found that deletion of *TET1* promoted the development of B cell non-Hodgkin lymphoma (B-NHL). TET1 appeared to be required for maintenance of the normal abundance and distribution of 5hmC, which prevented hypermethylation of DNA, and for regulation of the B cell lineage and of genes encoding molecules involved in chromosome maintenance and DNA repair. Then, they reasoned that based on these data, TET1-deficient B cell lymphomas might have specific mutations. Whole-exome sequencing (WES) indeed confirmed that these mutations were frequently found in B-NHL in which TET1 was hypermethylated and transcriptionally silenced. They conclude that *TET1* acts as a tumour suppressor of B-NHL. A more inventory approach was chosen by Braggio et al. [2] who performed a genome-wide analysis of 19 primary diffuse large B cell lymphomas (DLBCLs) of the central nervous system (CNS) by array comparative genomic hybridization (CGH) and WES. They found biallelic inactivation of TOX and PRKCD, which is not present in systemic DLBCL. In addition, they found a high prevalence of *MYD88* mutations (79 %) and *CDKN2A* biallelic loss (60 %) and alterations in many other genes of the NF-κB pathway, but these are not unique for DLBCL of the CNS. Overall NF-κB pathways were altered in >90 % of these cases. These findings indicate that primary DLBCL of the CNS genetically differs from DLBCL from other sites, but that there are also commonly affected pathways. A similar approach (using large amounts of data from multiple approaches on a rare tumour) was taken by Lee et al. [3] for 34 cases of extranodal NK/T cell lymphoma nasal type (ENKL). They performed multiple types of next-generation sequencing, including WES (9 cancer tissues and 4 cancer cell lines), targeted sequencing (21 cancer tissues) and RNA sequencing (3 cancer tissues and 4 cancer cell lines). Mutations were found most frequently in three genes, *STAT3*, *BCOR* and *MLL2* (which were present in nine, seven and six cancer samples, respectively), whereas there were only two cases of Janus kinase 3 (*JAK3*) mutation. In total, JAK/STAT pathway- and histone modification-related genes accounted for 55 and 38 % of the cancer samples, respectively, and their involvement in ENKL pathogenesis was also supported by gene expression analysis. They conclude that they found several novel driver genes of ENKL, which may be future therapeutic targets in this disease. Finally, with the present approach to sequencing, also more data regarding so-called pseudogenes become available and it has become clear that these may have more important roles than previously thought. Karreth et al. [4] show that mice engineered to overexpress either the full-length murine *B-Raf* pseudogene *Braf-rs1* or its pseudo ‘CDS’ or ‘3′ UTR’ develop an aggressive malignancy resembling human DLBCL. They show that *Braf-rs1* and its human ortholog, *BRAFP1*, elicit their oncogenic activity, at least in part, as competitive endogenous RNAs (ceRNAs) that elevate BRAF expression and MAPK activation in vitro and in vivo. Furthermore, they show that transcriptional or genomic aberrations of *BRAFP1* occur frequently in multiple human cancers, including B cell lymphomas. These data indicate that indeed also *pseudogenes* are relevant players in the development of B cell lymphomas.

All in all, these studies show that we seem to be only at the beginning of understanding the complexity of the molecular basis of cancer and specifically lymphomas.

### B cell lymphomas

One of the important drivers of B cell lymphomas, especially low-grade types, is the signalling though the antigen receptor. Many studies have addressed the composition of the immunoglobulin genes to get a better grip on the understanding of its role. Berget et al. [5] investigated the V family usage in 99 cases of low-grade follicular lymphoma (FL) and correlated the results with outcome. They found preferred usage of immunoglobulin heavy variable 3 (IGHV3) (58 %), and those patients with IGHV5 or more than one V family usage had a significantly worse outcome. In contrast with chronic lymphatic leukaemia (CLL), there was no difference in the prognosis between patients who had unmutated versus mutated sequences. These results indicate that the specific antigen receptor is probably quite relevant, but that more detailed understanding of the antigen (if any) involved is urgently needed. An example of a much more detailed approach is the work by Green et al. [6], who analyzed purified FL cells and identified additionally recurrently mutated genes and confirmed mutations of one or more chromatin modifier genes in almost all cases. They defined the hierarchy of somatic mutations arising during tumour evolution by analyzing the phylogenetic relationship of somatic mutations across the coding genomes of 59 sequentially acquired biopsies from 22 patients. *CREBBP* mutations were most significantly enriched within the earliest progenitor cell. These mutations were associated with a signature of decreased antigen presentation characterized by reduced transcript and protein abundance of MHC class II on tumour B cells, in line with the role of CREBBP in promoting class II transactivator (CIITA)-dependent transcriptional activation of these genes. *CREBBP* mutant B cells stimulated less proliferation of T cells in vitro compared with wild-type B cells from the same tumour. Transcriptional signatures of tumour-infiltrating T cells were indicative of reduced proliferation, and this corresponded to decreased frequencies of tumour-infiltrating CD4 helper T cells and CD8 memory cytotoxic T cells. These observations therefore implicate *CREBBP* mutation as an early event in FL evolution that contributes to immune evasion via decreased antigen presentation.

Further proof of the role of antigens as a driver of B cell lymphoma, in this case mantle cell lymphoma (MCL), comes from Xochelli et al. [7]. They found the AID full-length transcript and the most frequent splice variants (AID-ΔE4a, AID-ΔE) in 128 (96 %), 96 (72 %) and 130 cases (98 %) of MCL, respectively. Higher AID full-length transcript levels were significantly associated with lack of somatic hypermutation within the IGHV genes. Although most cases exhibited low levels of intraclonal diversification, analysis of the mutational activity revealed precise targeting of somatic hypermutation indicative of an active, ongoing interaction with antigen(s). These findings indeed strongly allude to antigen involvement in the natural history of MCL.

Pan et al. [8] hypothesized that epigenetic changes are important in the progression of DLBCL. They compared the genome-wide methylation profile of relapsed DLBCL with that of the original tumour and found a ‘relapse-associated methylation signature’ that included the transforming growth factor beta (TGF-β) receptor pathway. They also observed decreased intra-tumour methylation heterogeneity from diagnosis to relapsed tumour samples. Relapse-free patients display lower intra-tumour methylation heterogeneity at diagnosis compared with relapsed patients in an independent validation cohort. A better understanding of the underlying mechanism may therefore be of help to get a better idea on tumour progression in DLBCL.

A more targeted approach was taken by Dubois et al. [9] with a focus on enhancer of zeste homolog 2 (EZH2), of which recurrent somatic heterozygous gain-of-function mutations of *EZH2* have been identified in DLBCL. Since EZH2 inhibitors are being tested in phase 1 and 2 clinical trials, they want to develop a biomarker that predicts effect. By immunohistochemistry, they determined the methylation profiles of the DLBCL from 82 patients as well as the mutational profiles of *EZH2* in the lymphomas of 32 patients with DLBCL with a next-generation sequencing (NGS) approach (panel of 34 genes involved in lymphomagenesis). They developed a score based on H3K27me2 and H3K27me3 expression that distinguished patients with wild-type (WT) *EZH2* and patients with *EZH2* Y641 mutation. NGS analysis revealed a subclonal *EZH2* mutation pattern in *EZH2* mutant patients with WT-like immunohistochemistry (IHC) methylation profiles, while associated mutations capable of upregulating EZH2 were detected in WT EZH2 patients with mutant-like IHC methylation profiles. The next step is going to show that this score is really predicting therapeutic response of EZH inhibitors.

### T cell lymphoma

Cresenzo et al. [10] focus with their genomic evaluation on anaplastic lymphoma kinase (ALK)-negative (ALK−) anaplastic large cell lymphoma (ALCL). They identified activating mutations of *JAK1* and/or *STAT3* genes in approximately 20 % of 155 ALK− ALCLs and demonstrated that 38 % of systemic ALK− ALCLs displayed double lesions. Recurrent chimeras combining a transcription factor (*NFkB2* or *NCOR2*) with a tyrosine kinase (*ROS1* or *TYK2*) were also discovered in WT JAK1/STAT3 ALK− ALCL. All these aberrations lead to the constitutive activation of the JAK/STAT3 pathway, which was proved to be oncogenic. Finally, they showed that JAK/STAT3 pathway inhibition impairs cell growth in vitro and in vivo, and this is therefore a rationale for a new treatment approach.

## Epidemiology of lymphoma

Normally, we deal with the diagnosis of lymphomas and have good ideas on the treatments that will follow our diagnosis as well as the resulting outcomes. We even are aware of secondary cancers, which is especially a burden in survivors of Hodgkin lymphoma (HL). I think it is also relevant to have an idea of less visible consequences. Glimelius et al. [11] analyzed data from a population-based registry from Sweden on almost 2000 HL patients, with clinical data of more than 1000 of them, and compared these with those of a matched cohort. The data include patients diagnosed between 1992 and 2009 with follow-up until 2013. The results indicate that HL survivors have a 30–50 % increase in the risk of work loss, even when this was adjusted for secondary effects like cardiovascular disease and secondary cancers. This underpins the major effect a cancer diagnosis has for patients. This is an area that may be subject of overdiagnosis, and thus, major unnecessary impact is in paediatric cases, especially paediatric nodal marginal zone B cell lymphoma (NMZL). Excellent work from Kluin et al. [12] showed that the spectrum of paediatric marginal zone hyperplasia to lymphoma may have a common cause—a *Haemophilus influenzae*-driven immune response. In six children with polyclonal marginal zone hyperplasia and four adolescents with clonal NMZL, they looked for the presence of *H. influenzae*. In all patients with hyperplasia, they detected *H. influenzae* (either direct culture or highly sensitive PCR), whereas this was lacking in the lymphoma cases. In only 1/28 control samples, they detected the microorganism, in very low quantity. Of note, in the hyperplasia cases, they detected areas with light chain restriction (confirmed by flow cytometry). Since several *H. influenzae* strains are known to interact with the constant part of IgD on human B cells, leading to their polyclonal proliferation and activation, the authors speculate that in vivo stimulation of IgD+ marginal zone B cells by this bacterium may be implicated in this particular lymphadenopathy. The results of course indicate to be very careful with the diagnosis of paediatric NMZL; the methods they used to detect *H. influenzae* require quite some expertise and preferably the availability of frozen tissue and is therefore not feasible for most laboratories.

There are other microorganisms involved in lymphomas like the Epstein-Barr virus (EBV), hepatitis C and *Helicobacter pylori* (amongst others). According to Tognon et al. [13], we need to add simian virus 40 (SV40) to the list. In serum samples from NHL-affected patients (*n* = 150) along with controls represented by patients with other cancers (*n* = 142) and healthy subjects (*n* = 300), they found antibodies against SV40 in about 40 % of the NHL patients, 20 % in other cancer patients and 15 % in healthy individuals. Of course, such an association does not directly proof that SV40 plays a role in the lymphoma development, but is interesting enough to further study its role.

To have an idea on the outcome of rare lymphomas, there is a need for data from large registries. Xing et al. [14] analyzed clinicopathologic features for 107 patients (diagnosed between 1985 and 2012) with splenic MZL (SMZL) which accounts for less than 2 % of all NHL cases. The median age was 67 years (range 30–88), with 40 % male, almost all stage IV and with splenomegaly, bone marrow involvement and peripheral blood involvement (87 %). As initial treatment, 52 underwent splenectomy (10 with chemotherapy), 38 chemotherapy alone (21 chemoimmunotherapy containing rituximab, 1 rituximab alone) and 2 antivirals for hepatitis C, and 15 were observed only. The 10-year overall survival for first-line splenectomy versus chemotherapy was 61 and 42 %, respectively. The 10-year failure-free survival after first-line splenectomy versus chemotherapy was 39 and 14 %. Amongst the 38 patients who received first-line chemotherapy, the addition of rituximab did not result in increased survival. Fifteen patients transformed to aggressive lymphoma with median time to transformation of 3.5 years (range 6 months to 12 years), and the 10-year transformation rate was 18 %. The authors conclude that splenectomy remains a reasonable treatment for patients with SMZL. Parry et al. [15] try to find prognostic markers for this lymphoma type. In 175 cases, they found recurrent mutations in *TP53* (16 %), *KLF2* (12 %), *NOTCH2* (10 %), *TNFAIP3* (7 %), *MLL2* (11 %), *MYD88* (7 %) and *ARID1A* (6 %). *KLF2* mutations were early, clonal events, enriched in patients with del(7q) and IGHV1-2*04 B cell receptor immunoglobulins, and were associated with a short median time to first treatment. In multivariate analysis, mutations in *NOTCH2* and 100 % germline *IGHV* gene identity were independent markers of short time to first treatment, while *TP53* mutations were an independent marker of short overall survival. They conclude that they have demonstrated that *NOTCH2* and *TP53* gene mutations are independent markers of reduced treatment-free and overall survival in SMZL. How this translates to altered clinical decision-making remains unclear.

Litvinov et al. [16] investigated the geographic clustering of cutaneous T cell lymphoma (CTCL), which is always quite risky in rare tumours. Using region, zip code, age, sex and ethnicity, the authors analyzed the demographic data of 1047 patients from Texas from 2000 through 2012 and another set of 1990 patients who were recorded between 1996 and 2010. There was geographic clustering of patients in three communities in which CTCL incidence rates were 5 to 20 times higher than the expected population rate. The authors conclude that their data indicate the existence of yet unknown external causes/triggers for this rare malignancy. Although the data are relatively large, still such conclusions have to be drawn very cautiously, since familial clustering, enhanced awareness and chance may underlie such clustering as well.

## Defining entities

### B cell lymphomas

A mutation in the *MYD88* gene is present in over 90 % of lymphoplasmacytic lymphomas (LPLs) and since this entity is sometimes difficult to separate from MZ Martinez-Lopez et al. [17] investigated lymphoma samples from 19 patients with LPL, 88 patients with SMZL, 8 patients with nodal (N)MZL and 21 patients with extranodal (EN)MZL. Of note, upon review and integrating mutational, histologic and clinical data, five cases were reclassified as LPL, reiterating the fact that this diagnosis is not always straightforward. After reclassification, *MYD88* L265P was detected in 13/86 (15 %) SMZL and in 19/24 LPL (79 %) cases. The mutation was absent from NMZL and ENMZL cases. A strong correlation was found between the presence of an IgM monoclonal paraproteinaemia and the *MYD88* L265P mutation. SMZL cases positive for *MYD88* L265P were also associated with monoclonal IgM paraproteinaemia (4/13 cases), although with less serum paraproteinaemia. They also had a higher frequency of plasmacytic differentiation (9/13) but with no correlation between the presence of mutation and of light chain-restricted plasma cells in tissue. The authors conclude that demonstration of the *MYD88* L265 mutation is a valuable tool for the diagnosis of LPL, although some SMZL cases carrying the mutation do not fulfil the diagnostic criteria for LPL. The *MYD88*-negative LPL cases, nevertheless, may pose a challenge to separate from NMZL with plasmacytic differentiation.

Morita et al. [18] addressed the issue of so-called in situ FL. As I discussed in previous reviews of the literature, this lesion is rare in unselected patient groups and seems to have little clinical consequences when found as an isolated finding. The approach of Morita et al. was the other way around: they looked for patients with FL who had a previous abdominal cancer with lymph node resection and found 4 such patients from a total of 150 patients with FL. All four had in situ FL in the previously resected lymph nodes. The time from lymphadenectomy to the diagnosis of FL was 23–120 months. They conclude that although the rate for development of FL in individuals with in situ FL is low in prospective studies, their data indicate that follow-up studies for a longer period are necessary. However, I would certainly not advocate to look for this lesion with bcl2 staining in lymph nodes from abdominal cancer patients, since it would result in unnecessary worry of a second malignancy in these cancer patients while it still seems that the chance that they have clinical relevant disease is low and there is no place for early treatment.

Some FL cases are CD5 positive, but the meaning of this aberrant expression is not clear. Li et al. [19] collected 88 such cases (53 men and 35 women; median age, 60 years; range, 31–86). The lymphoma was diagnosed relatively often at an extranodal (EN) site (initially in lymph nodes in 66 and EN sites in 22 patients). The presence of t(14;18)(q32;q21)/*IGH-BCL2* or another *BCL2* translocation was relatively low in 28/44 (64 %) cases. Clinical features also differed with CD5-negative FL; with a median follow-up of 55 months, 15 patients died which is rather high; this fits with the relatively high International Prognostic Index, and commonly development of DLBCL (38/88). They conclude that CD5 expression in FL is associated with an adverse outcome; however, it remains questionable whether all these cases really represent FL or may be other lymphoma types with extensive follicular colonization.

Primary DLBCL of the testis is a lymphoma with specific clinical and pathological features. Twa et al. [20] performed bacterial artificial chromosome capture sequencing on three such cases and found novel *CIITA*, *FOXP1* and *PDL* rearrangements involving *IGHG4*, *FLJ45248*, *RFX3*, *SMARCA2* and *SNX29*. Immunohistochemistry showed an association between *PDL* rearrangements and increased protein expression. Using fluorescence in situ hybridization (FISH) in a larger series of testicular DLBCL, *CIITA* (8/82; 10 %) and *FOXP1* (5/74; 7 %) rearrangements appeared to be recurrent. These data suggest that immune-checkpoint inhibitor therapy might be a promising intervention for this rare lymphoma type that develops in an immune-privileged site.

### T cell lymphomas

ALK-positive and ALK-negative ALCL share morphological features but differ in prognosis and, obviously, ALK expression as a result from the t(2;5) translocation. Steinhilber et al. [21] compared the microRNA (miRNA) expression between these two types. Their NGS approach identified 106 significantly differentially expressed miRNAs. The top significantly differentially expressed miRNAs included five upregulated miRNAs: miR-340, miR-203, miR-135b, miR-182 and miR-183 and seven downregulated miRNAs: miR-196b, miR-155, miR-146a, miR-424, miR-503, miR-424* and miR-542-3p. The miR-17-92 cluster was also upregulated in ALK+ cells. Additionally, they identified a signature of three miRNAs significantly regulated by the transcription factor C/EBPβ, which is specifically overexpressed in ALK+ ALCL, including the miR-181 family. Of interest, miR-181a, which regulates T cell differentiation and modulates T cell receptor (TCR) signalling strength, was significantly downregulated in ALK+ ALCL cases. These data reveal a miRNA signature linking ALK+ ALCL to a deregulated immune response and may reflect the abnormal TCR antigen expression known in ALK+ ALCL. Merkel et al. [22] take this work further. They also found that ALK-positive ALCL, in contrast to ALK-negative ALCL, displays low miR-155 expression, and discovered that this is due to miR-155 promoter methylation, but there was no direct effect of the ALK kinase on miR-155 levels. Induced overexpression of miR-155 in ALK-positive ALCL cell lines resulted in reduced levels of *C/EBPβ* and *SOCS1*. In murine engraftment models of ALK− ALCL, anti-miR-155 mimics are able to reduce tumour growth and increase levels of cleaved caspase-3 and SOCS1 resulting in suppression of STAT3 signalling. These data suggest that miR-155 can act as a tumour driver in ALK-negative ALCL, and blocking miR-155 could be therapeutically relevant. The data also indicate that ALK-positive ALCL and ALK-negative ALCL are biologically really different entities.

### Cutaneous lymphomas

Cutaneous follicle centre cell lymphoma (CFCL) is regarded as a completely different disease than FL, although FL may be manifested in the skin. Several studies have shown that CFCL lacks a *bcl2* rearrangement in contrast to FL. Pham-Ledard et al. [23] confirm this knowledge by analyzing 47 CFCL, 4 of which carry a *bcl2* break by FISH, and 6 FL presenting with skin involvement, 3 of which have a *bcl2* break. Since some of the CFCL with a *bcl2* break had systemic disease and poor prognosis, one may doubt the diagnosis in these cases.

## Pitfalls in lymphoma diagnosis

The role of EBV in lymphoproliferation in patients with various forms of immune suppression is well known, but it remains a challenge when to investigate for EBV and what to do with a positive result. In patients with inflammatory bowel disease (IBD), several case reports indicate an increased risk in EBV-positive lymphoproliferations, but it is unclear how aggressive this disease is. Nissen et al. [24] collected biopsies from 58 patients with IBD who had been tested for the presence of EBV, 28 of which were positive. The presence of EBV was correlated with an atypical infiltrate, which was present in more than 50 % of the EBV-positive cases and only one of the EBV-negative cases. Two EBV-positive patients had overt lymphoma, and eight had a monomorphic infiltrate. After reducing immune suppression, the morphology returned to normal, although one of the two lymphoma patients had in addition chemotherapy. No patient died from the lymphoproliferation, but four EBV-positive patients underwent colectomy due to severe complaints. These data indicate that the presence of EBV-positive lymphoproliferation in IBD can be suspected based on morphology, but that it is less aggressive compared to other immune-suppressed situations.

## Prognostic factors in lymphoma

Also this time, there is a small selection of articles that deal with prognostication in NHL. I chose those articles that have high-quality data, including a discovery and a confirmation cohort and well-defined relevant patient selection.

In FL, Kridel et al. [25] applied IHC for CD68 and CD163 to two large tissue microarrays (TMAs): one with samples from 186 patients from the BC Cancer Agency (BCCA) and the other with 395 samples from the PRIMA trial patients. Both groups had been treated with rituximab, but within different chemotherapy regimes. Macrophage infiltration was assessed using image analysis, and an increased CD163-positive pixel count was predictive of an adverse outcome in the BCCA dataset (5-year progression-free survival 38 versus 72 % in the training cohort and 29 versus 61 % in the validation cohort). However, in the PRIMA trial, an increased CD163 pixel count was associated with favourable outcome (60 versus 44 % in the training cohort and 55 versus 37 % in the validation cohort). These data clearly indicate the importance to correlate a prognostic factor to well-defined patient groups, but also that it is difficult to introduce such a marker in clinical practice.

miRNA profiling is very well feasible on paraffin-embedded tissue specimens, and therefore, many studies are done using this technique. A good example is the work by Husby et al. [26] who performed genome-wide miRNA microarray profiling of 74 diagnostic MCL samples from the Nordic MCL2 trial (screening cohort). Prognostic miRNAs were validated in diagnostic MCL samples from 94 patients of the independent Nordic MCL3 trial (validation cohort). Three miRNAs (miR-18b, miR-92a and miR-378d) were significantly differentially expressed in patients who died of MCL in both cohorts. miR-18b was superior to miR-92a and miR-378d in predicting high risk. Transfection of two MCL cell lines with miR-18b decreased their proliferation rate without inducing apoptosis, suggesting that miR-18b may render MCL cells resistant to chemotherapy by decelerating cell proliferation. Based on these results, they generated a new biological MCL International Prognostic Index (MIPI-B)-miR prognosticator, which improved the identification of high-risk patients with regard to cause-specific, overall and progression-free survival.

The lymphoma on which most prognostic work is done is DLBCL. Shepshelovich et al. [27] performed miRNA profiling but had only 83 samples, predefined in favourable and poor prognosis, with a validation set of only 13 cases. The approach resulted in significant differences, but the findings did not add to the standard prognostic indicators. Knudsen et al. [28] took a more thorough approach. They developed a miRNA response predictor in DLBCL based on the measured growth inhibition of 60 human cancer cell lines (NCI60) in the presence of doxorubicin, cyclophosphamide, vincristine and etoposide as well as the baseline microRNA expression of the 60 cell lines. This response predictor consisting of 20 microRNAs was blindly validated in a cohort of 116 de novo DLBCL patients treated with R-CHOP or R-CHOEP as first-line treatment. The predicted sensitivity based on diagnostic FFPE samples matched the clinical response. When the International Prognostic Index (IPI) was included in the prediction analysis, the separation between responders and non-responders improved. The predictions were almost the same when diagnostic biopsies were used as when relapse biopsies were used. These preliminary findings warrant testing in a larger cohort of relapse patients to confirm whether the miRNA-based predictor can select the optimal second-line treatment and increase survival.

*MYC* status is a relevant issue in DLBCL, but the impact varies amongst the subtype of lymphoma and the type of MYC alteration. Caponetti et al. [29] investigated the significance of *MYC*, *BCL2* and *BCL6* gene abnormalities in a cohort of 205 DLBCL patients studied by conventional and/or fluorescence in situ hybridization cytogenetic analysis. One hundred seventy-two cases (84 %) were classified as *MYC*−, 17 (8 %) were *MYC*+/*BCL2*−/*BCL6*−, and 16 (8 %) were double/triple-hit lymphomas (i.e. *MYC*+/*BCL2*+, *MYC*+/*BCL6*+ or *MYC*+/*BCL2*+/*BCL6*+). They found a significant difference in event-free survival amongst the three groups, with the double/triple-hit group having the worst outcome. Somewhat surprising, patients who were *MYC*+, but *BCL2*− and *BCL6*−, do not have a worse outcome when compared to those who are *MYC*− which is in contrast with existing data in the literature. Li et al. [30] looked for MYC protein expression in primary mediastinal DLBCL, although these cases rarely have a genetic event that includes a *MYC* alteration. They found variable MYC protein expression by IHC in 30 (94 %) of 32 cases including 10 cases with high MYC IHC expression of at least 30 % positive nuclei. FISH analyses for *MYC* rearrangement on these 10 cases were negative. Review of clinical data of a subset (!) of cases with high- and low-MYC IHC expression showed no differences in clinical outcome. These results are somewhat difficult to interpret, but actually, the numbers are too low to draw meaningful conclusions. Even the work of Wang et al. [31], which has findings that are contradictory to these results, lacks sufficient power, although they study 135 cases of DLBCL. They analyzed the differences between genetic double-hit lymphomas and those defined on protein expression. They determined in their cohort in whom *MYC*/8q24 and *BCL2*/t(14;18)(q32;q21) statuses were assessed by FISH at diagnosis of MYC and BCL2 expression by immunohistochemistry. A total of 54 (40 %) cases were positive for both MYC and BCL2, supporting a diagnosis of double-hit lymphoma, which is a quite high number compared to those of the literature. Amongst them, 19 (35 %) had *MYC* rearrangement, 12 (22 %) had multiple copies of *MYC*, 19 had no *MYC* abnormalities, and in 4 cases, FISH analysis failed. *BCL2* abnormalities were present in 28/54 (52 %) cases (20 rearranged and 8 multiple copies). *MYC* rearrangement correlated with a significantly worse overall survival, whereas *BCL2* genetic status did not correlate with survival. They conclude that MYC and BCL2 expression by immunohistochemistry correlates with gene status by FISH, but that immunohistochemistry is neither specific nor adequately sensitive to be used as a surrogate for *MYC* and *BCL2* gene status using any cut-off level.

Enteropathy-associated T cell lymphoma (EATL) has a particularly poor prognosis, and the International Prognostic Index and the prognostic index for peripheral T cell lymphoma have limited predictive value for outcome. De Baaij et al. [32] performed a retrospective multicenter study, based on 92 patients. They were able to distinguish three risk groups: a high-risk group, characterized by the presence of B symptoms (median overall survival of 2 months); an intermediate-risk group, comprising patients without B symptoms and an IPI score ≥2 (7 months); and a low-risk group, representing patients without B symptoms and an IPI score of 0–1 (34 months). They conclude that their new validated prognostic model (EPI) accurately predicts survival outcome in EATL and may be used for patient selection for new therapeutic strategies and evaluation of clinical trials.

## Ancillary techniques

Technological development is one of the drivers of modern pathology. New methods therefore need to be evaluated all the time to see whether they can improve standard pathology. Liang et al. [33] retrospectively analyzed EBV status in 232 DLBCL patients using EBV-encoded small RNA (EBER) in situ hybridization (ISH) and EBV DNA analysis in whole blood. EBER was positive in 24 (10 %) patients, and EBV DNA was positive in 18 (10 %) patients; the two analyses had more than 90 % concordance. EBV-positive patients as determined by both techniques had worse overall survival than those without EBV positivity. The additional value of blood testing above tissue testing lies in the following: the transformation from positive to negative after cycle 3 with chemotherapy had the most capacity to distinguish a superior from an inferior outcome. These findings suggest that EBV DNA in whole blood has good concordance with EBER ISH and that it may be a good biomarker for monitoring the disease.

Although clonality testing is already a high level, high-quality test, the development of next-generation sequencing may have its impact in this area of pathology, too. Appenzeller et al. [34] developed a next-generation sequencing approach on the ion torrent personal genome machine to characterize the immunoglobulin heavy gene V-D-J rearrangements. The method was applied to two diagnostic tissue samples, including formalin-fixed and paraffin-embedded tissue, of two patients with iatrogenic immunodeficiency-associated Epstein-Barr virus lymphoproliferative disorder, with ulcerative colitis as underlying disease. The immunoglobulin rearrangement sequences obtained by next-generation sequencing revealed undoubtedly clonally related lesions in two tissue biopsies that were taken over time in the first patient, which is concordant with disseminated lymphoma. The other patient showed two clonally unrelated lesions, which is incompatible with clonal dissemination. This information was not inferred from evaluation of the heavy and light chain rearrangements by fragment analysis, which is currently the gold standard. This study nicely demonstrates the diagnostic application of next-generation sequencing of immunoglobulin rearrangement assessment in pathology for clinical decision-making in patients with several simultaneous or subsequent lymphoproliferations. Another study using a next-generation sequencing approach investigated a different potential use. Kurtz et al. [35] prospectively evaluated its utility in 311 blood and 105 tumour samples from 75 patients with DLBCL, comparing the cellular (circulating leukocytes) and acellular (plasma cell-free DNA) compartments of peripheral blood. The results were related to clinical outcomes and 18FDG PET/CT (*n* = 173). Clonal immunoglobulin rearrangements were detected in 83 % of patients with adequate tumour samples to enable subsequent monitoring in peripheral blood (which is lower than when the complete Biomed set is used). Molecular disease measured from plasma, as compared to circulating leukocytes, was more abundant and more correlated with radiographic disease burden. Prior to treatment, molecular disease was detected in the plasma of 82 % of patients compared to 71 % in circulating cells. However, molecular disease was detected significantly more frequently in the plasma at time of relapse (100 vs. 30 %). Detection of molecular disease in the plasma often preceded PET/CT detection of relapse in patients initially achieving remission. This method, therefore, is promising, as confirmed by Roschewski et al. [36] who performed similar work. Clonal products were identified in pretreatment specimens from 126 patients who were followed up for a median of 11 years. Interim monitoring of circulating tumour DNA at the end of two treatment cycles in 108 patients showed a 5-year time to progression of about 40 % in patients with detectable circulating tumour DNA and 80 % in those without detectable circulating tumour DNA. So, also this study indicates that surveillance of circulating tumour DNA identifies patients at risk of recurrence before clinical evidence of disease.

Already more than 10-year DLBCL is subdivided into germinal centre B cell-like and activated B cell-like subtypes. Still, these lymphomas are difficult to differentiate in routine diagnosis, impeding the use of this subtyping in clinical decision-making. Mareschal et al. [37] therefore developed a simple and rapid classifier based on a reverse transcriptase multiplex ligation-dependent probe amplification assay and 14 gene signatures. Compared with the Affymetrix U133 + 2 gold standard, all 46 samples of a validation cohort classified using both techniques were attributed to the expected subtype. Similarly, 93 % of the 55 samples of a second independent series characterized with a mid-throughput gene expression profiling method were classified correctly. The developed assay was sensitive enough to obtain reliable results from formalin-fixed, paraffin-embedded samples and flexible enough to include prognostic factors such as MYC/BCL2 co-expression. Finally, in a series of 135 patients, both overall and progression-free survival differences between the two subtypes were confirmed. As the authors conclude, because the multiplex ligation-dependent probe amplification method is already in use and requires only common instruments and reagents, it could easily be applied to clinical trial patient stratification to help in treatment decisions.
